# Data-Driven Analysis of Antimicrobial Resistance in Foodborne Pathogens from Six States within the US

**DOI:** 10.3390/ijerph16101811

**Published:** 2019-05-22

**Authors:** Nina Zhang, Emily Liu, Alexander Tang, Martin Cheng Ye, Kevin Wang, Qian Jia, Zuyi Huang

**Affiliations:** 1 Wissahickon High School, Ambler, PA 19002, USA; nzhang2010@aol.com; 2 North Penn High School, Lansdale, PA 19446, USA; liuemily123@gmail.com (E.L.); martinch.ye@gmail.com (M.C.Y.); 3 Germantown Academy, Fort Washington, PA 19034, USA; alextang1818@gmail.com; 4 Lower Moreland High School, Huntingdon Valley, PA 19006, USA; Kevinwang5181@gmail.com; 5 Department of Health, Nutrition & Exercise Sciences, Immaculata University, Immaculata, PA 19345, USA; qjia@Immaculata.edu; 6 Department of Chemical Engineering, Villanova University, Villanova, PA 19085, USA

**Keywords:** foodborne pathogens, antimicrobial-resistance genes, principal component analysis, hierarchical clustering

## Abstract

Foodborne pathogens cause thousands of illnesses across the US each year. However, these pathogens gain resistance to the antimicrobials that are commonly used to treat them. Typically, antimicrobial resistance is caused by mechanisms encoded by multiple antimicrobial-resistance genes. These are carried through pathogens found in foods such as meats. It is, thus, important to study the genes that are most related to antimicrobial resistance, the pathogens, and the meats carrying antimicrobial-resistance genes. This information can be further used to correlate the antimicrobial-resistance genes found in humans for improving human health. Therefore, we perform the first multivariate statistical analysis of the antimicrobial-resistance gene data provided in the NCBI Pathogen Detection Isolates Browser database, covering six states that are geographically either in close proximity to one another (i.e., Pennsylvania (PA), Maryland (MD), and New York (NY)) or far (i.e., New Mexico (NM), Minnesota (MN), and California (CA)). Hundreds of multidimensional data points were projected onto a two-dimensional space that was specified by the first and second principal components, which were then categorized with a hierarchical clustering approach. It turns out that *aadA*, *aph(3’’)*, *aph(3’’)-Ib*, *aph(6)-I*, *aph(6)-Id*, *bla*, *blaCMY*, *tet*, *tet*(A), and *sul2* constructed the assembly of ten genes that were most commonly involved in antimicrobial resistance in these six states. While geographically close states like PA, MD and NY share more similar antimicrobial-resistance genes, geographically far states like NM, MN, and CA also contain most of these common antimicrobial-resistance genes. One potential reason for this spread of antimicrobial-resistance genes beyond the geographic limitation is that animal meats like chicken and turkey act as the carriers for the nationwide spread of these genes.

## 1. Introduction

Across the US, foodborne pathogens cause illness in approximately 48 million people each year and impose over a $15.5 billion economic burden annually [1]. In particular, there are 31 pathogens known to cause foodborne illness [2]. Just with these 31 pathogens alone, there are an estimated 9.4 million illnesses annually, leading to estimated 55,961 hospitalizations and 1351 deaths a year (90% credible interval) in the US [3]. These pathogens obtain antimicrobial-resistance genes and become resistant to existing antimicrobials, encoding proteins with antimicrobial-resistance functions. Specifically, these proteins degrade antimicrobials, pump antimicrobials out of the cells, or change the active binding sites for antimicrobials [4]. Each year in the US, at least 2 million people become infected with antimicrobial-resistant bacteria and at least 23,000 people die as a direct result [5]. Bacteria have mobile elements that can be transferred between different bacteria (such as plasmids that contain antimicrobial-resistance genes) and thus are released into the environment for another bacterium to take. This is known as horizontal gene transfer [6]. Foodborne pathogens generally obtain multiple antimicrobial-resistance genes, which equip the pathogens with multiple resistance functions (e.g., antimicrobial degradation, antimicrobial binding site alteration, and antimicrobial efflux pump). This enables foodborne pathogens to resist multi-antimicrobials [7]. Antimicrobial-resistance genes are spread by pathogens that are carried in foods (e.g., meats). In particular, farm animals carry bacteria in their intestines and are given antimicrobials frequently. Overdoses of oral prescription of antimicrobials for animals will destroy or inhibit part of their intestinal bacteria, but the overuse of antimicrobials may cause the mutation that enables bacteria to survive and multiply. These bacteria, which carry antimicrobial-resistance genes, go forth to contaminate meats and other animal products during the slaughtering and further processing of the meat. The bacteria may also contaminate animal feed and drinking water through infected bodily fluids. These antimicrobial-resistant bacteria, along with the genes they carry, are then passed to people through industrial animal food production. It is thus important to study the genes that are most related to antimicrobial resistance and the pathogens/foods that carry them. 

Fortunately, antimicrobial resistance data in the US are actively collected through the National Database of Antibiotic Resistance Organisms (NDARO), the NCBI Pathogen Detection Isolates Browser (NPDIB), and the National Antibiotic Resistance Monitoring System (NARMS). Among these databases, only the NPDIB database shows the antimicrobial genes sampled from four types of meats (i.e., chicken, beef, pork, and turkey). While these databases are available, little research has been done to systematically analyze the data, study how antimicrobial-resistance genes are carried by pathogens and meats throughout the US, and identify the set of the most common antimicrobial-resistance genes. The NPDIB lists antimicrobial-resistance genes carried by pathogens that were isolated from patients, food, and environmental samples in state and federal laboratories over time. Founded in collaboration with the Food and Drug Administration (FDA), the Centers for Disease Control and Prevention (CDC), the United States Department of Agriculture (USDA), and other institutions, the NPDIB allows people to search for pathogen isolates and identify pathogens with particular antimicrobial-resistance genes. Since foodborne pathogens are sequenced and submitted to the NPDIB in real time, it allows for quick diagnosis and detection of pathogens that cause foodborne disease outbreaks. 

While the NPDIB database itself contains a significant amount of important information on foodborne pathogens and antimicrobials, few studies have been conducted to extract meaningful information from its gene data. The NPDIB database is typically used to detect pathogens by comparing the genomic sequences in it with the pathogens isolated from particular foods. On the other hand, there are papers that have analyzed data from the NARMS. For example, Sivapalasingam et al. in 2006 [8] utilized data from NARMS to study *Shigella* isolates in the US from 1999 to 2002. Since 1999, NARMS has tested every tenth *Shigella* isolate from 16 public health laboratories for susceptibility to 15 antimicrobials. That paper used the data from NARMS to confirm what percentage of *Shigella* was resistant and in which geographic regions these antimicrobials were most prevalent. However, the paper did not expound upon the meat industry. Another paper, Zhao et al. in 2009 [9] did focus on the meat industry and analyzed data from NARMS. However, the paper only focused on *Salmonella* and its resistance to antimicrobial agents from five beta-lactamase gene families. Although the findings indicated a varied spectrum of resistance present in *Salmonella* strains in the meat supply chain of the US, the paper did not analyze the geographical distribution of these meats and pathogens through the food industry. 

As mentioned above, little data analysis has been conducted to use those existing databases to extract useful information. In this work, we perform the first multivariate statistical analysis of gene data from the NPDIB database for six states that are geographically either close (i.e., PA, MD, and NY states) or far (i.e., NM, MN, and CA). The specific antimicrobial resistance found in these six states may direct the choice of antimicrobials used in these geographic areas. We aim to identify the antimicrobials to which pathogens show most resistance in these states, the genes that are mostly involved in antimicrobial resistance, and the carrying of antimicrobial-resistance genes via the pathogens and meats in these states. 

We study the impact of geographic location on the distribution of antimicrobial-resistance genes. Since each of the six states contains hundreds of samples of antimicrobial-resistant pathogens and over 100 antimicrobial-resistance genes, we implement principal component analysis (PCA) [10,11] to reduce the data dimensions so that we can visualize each dataset in a two-dimensional space. On the basis of the reduced data space characterized by PCA, hierarchical clustering is used to identify the antimicrobials, genes, pathogens, and meats that are mostly involved in the antimicrobial resistance. Hierarchical clustering is one of the most commonly used approaches for separating data points while providing similarity analysis between data points [12,13,14].

## 2. Materials and Methods

### 2.1. Data from the NCBI Pathogen Detection Isolates Browser (NPDIB)

Data from the NPDIB database for six states (including PA, MD, NY, NM, MN, and CA) from January 1970 to December 2018 were analyzed were analyzed in this project. The following six-dimensional information was obtained for each data sample: (1) the location (i.e., from which state the data were obtained); (2) the time (i.e., which year the data were sampled); (3) the food the data were sampled from (e.g., beef, chicken, turkey, and pork); (4) the foodborne pathogens detected in the sample; (5) the antimicrobial-resistance genes detected in the foodborne pathogens; and (6) the antimicrobials to which the detected foodborne pathogens are resistant. The data were generally obtained in the period between 1980 and December 2018 in the selected six states. While foodborne pathogens were also detected in foods other than in meats, such as fruits and vegetables, this project focused on four types of animals, including chicken, turkey, pork, and beef. This decision was based on findings that antimicrobial resistance is highly correlated to the abuse of antimicrobials in raising farm animals [15,16]. Over-crowded animals are raised on farms to improve the productivity of the meats [17]. Pathogens are thus easily passed from one animal to another. The stress from the overused antimicrobials is one potential force driving the evolution of pathogens [18]. Those pathogens surviving from the antimicrobial treatment contain antimicrobial-resistance genes and pass those genes to other pathogens. The pathogens are then delivered to soils and to other foods like fruits and vegetables. Therefore, analysis of the data sampled from meats may provide useful information for regulating the use of antimicrobials. Data for turkey, chicken, beef, and pork were selected as they were most the commonly sampled foods in all six states. The data were downloaded from the NPDIB webpage separately and saved in Excel as *.cvs documents for statistical analysis. The following were then identified from the data: foodborne pathogens, antimicrobial-resistance genes, the antimicrobials to which foodborne pathogens are resistant, and meats carrying antimicrobial-resistance genes. 

### 2.2. Multivariate Statistical Analysis: Principal Component Analysis and Hierarchical Clustering

**Figure 1:**
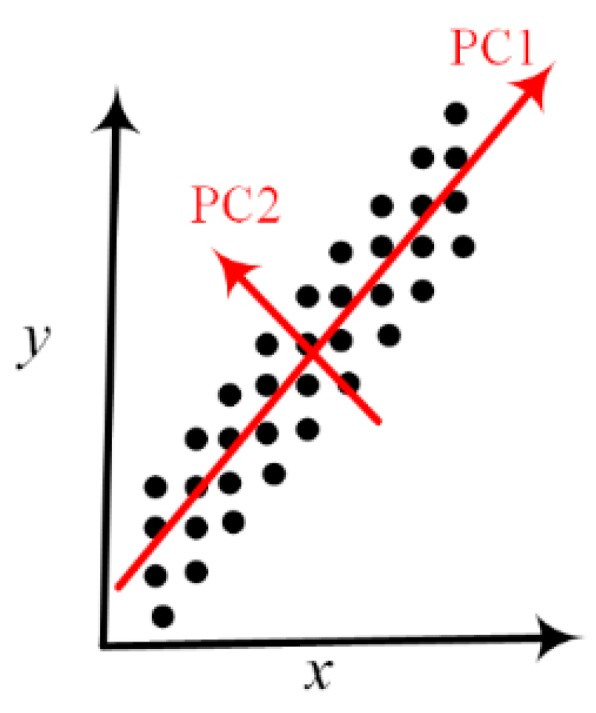
A schematic representation of principal component analysis. The data points contained in the dataset characterized by the original coordinate variables *x* and *y* are more distinguishable by their projections onto the PC1 direction than either *x* or *y* directions. The projections onto PC1 contain the largest variance, which suggests that the two-dimensional *x*–*y* space may be reduced to the one-dimensional PC1 space.

Each of the above-mentioned data points contained six dimensions: sampling location, sampling time, food source, foodborne pathogen, detected microbial-resistance gene, and antimicrobials to which foodborne pathogens become resistant. Hundreds of samples were obtained for each of the six states. In order to visualize the six-dimensional dataset, PCA was implemented to reduce the dimensions of the dataset and present it in a two-dimensional space. The basic idea of PCA is to identify new orthogonal coordinate directions upon which the projection of the original data has the largest variance (Figure 1). A larger variance means more information is retained in that projected coordinate direction. Compared to the original coordinate directions *x* and *y*, the first principal component (recorded as PC1) offers a better direction to distinguish the data points. Specifically, the overlaps of the projected data points onto PC1 is less than those onto either the direction *x* or *y*. In other words, PCA aims to identify new directions that can retain the most information from the original dataset. Typically, PC1 is the direction containing the largest variance in the data projections, followed by PC2. Both of these directions are the linear combinations of the original coordinate variables. The data projections onto PC1 and PC2 are generally used to reduce the original high-dimensional dataset in a two-dimensional space. Extensive studies have been conducted applying PCA so as to reduce a high-dimension dataset [19,20,21]. In this project, PCA was used to project the antimicrobial-resistance genes and the foodborne pathogens and meats that carry them onto a two-dimensional space specified by PC1 and PC2. For example, in order to analyze antimicrobial-resistance genes using PCA, the data containing the genes and the antimicrobials to which pathogens show resistance were constructed in a matrix in such a way that each row represents one gene while each column corresponds to one antimicrobial. The data matrix shows the total number of cases in which the gene (row) was detected in the microorganisms that resisted the antimicrobial (column) over all the years in the dataset. Each antimicrobial in the dataset thus represents one dimension to characterize the antimicrobial genes. Typically, there are more than ten antimicrobials to which pathogens show most resistance in a dataset. 

It is impossible to visualize antimicrobial-resistance genes in a ten-dimensional space without applying dimension-reduction approaches like PCA. The projection of the data onto the reduced-dimension space allows for the identification of outliers in the group of antimicrobial-resistance genes, which may contain important biological value. While PCA is effective in reducing the high-dimensional space to the PC1–PC2 space, each point of PC1 and PC2 is a linear combination of the original coordinate variables (i.e., the number of times of an antimicrobial getting resistance in this case) and loses a specific physical meaning. This may limit the application of PCA in mathematical modeling when people are interested in the physical meaning of model variables. However, it does not hinder the benefit of PCA to facilitate visualization of high-dimensional datasets. 

**Figure 2:**
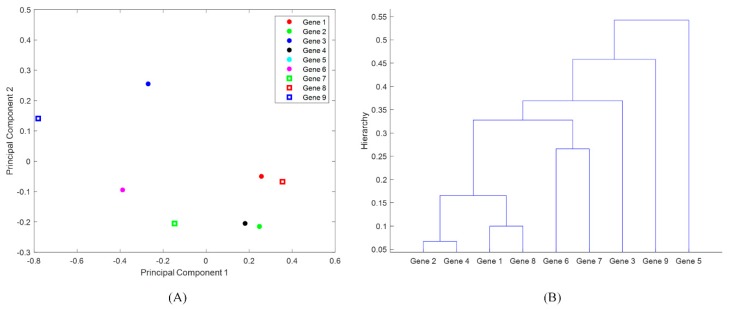
An illustrative example of hierarchical clustering: (**A**) nine genes are projected onto the PC1–PC2 space based on their resistance to antimicrobials azithromycin, cefoxitin, ceftiofur, ceftriaxone, chloramphenicol, ciprofloxacin, gentamicin, kanamycin, nalidixic acid, streptomycin, sulfisoxazole, tetracycline, and trimethoprim-sulfamethoxazole; (**B**) clustering the genes on the basis of their projections in (**A**).

In this project, PCA was performed in the software R, which is one of the most popular free programs in statistical data analysis [22]. While PCA provides an avenue for visualizing the high-dimensional dataset, it does not directly quantify the relationship between the data points. For example, although PCA can project the antimicrobial-resistance genes onto the PC1–PC2 space that is rotated from the space characterized by antimicrobials, it does not show how similar each of the two antimicrobial-resistance genes look. In particular, the data points shown in the PC1–PC2 space were difficult to separate on the plotted PCAs, especially when there were many overlapped data points (as was the case with genes). In order to address this issue, the hierarchical clustering approach was further applied here to study the similarity of the data points shown in the two-dimensional PC1–PC2 space. Figure 2 shows an illustrative example of hierarchical clustering. Certain antimicrobial-resistance genes were plotted in the PC1–PC2 space (Figure 2A). Gene 2 and Gene 4 are the closest to each other in Figure 2A, and they show most similar resistance patterns to the tested antimicrobials. These two genes then form one group, which then substitutes Genes 2 and 4 as new members for further clustering with other genes. The essential idea of hierarchical clustering was adding one item/group at a time to the closest points until all items in the dataset were included. For the dataset shown in Figure 2A, the result of hierarchical clustering is shown in Figure 2B. The lower the two genes are connected on the hierarchical tree (i.e., Figure 2B), the more similarly they are involved in the resistance to the selected antimicrobials (shown in the columns of the data matrix). For example, Genes 3, 5, and 9 are located quite far away from the other genes in Figure 2A, and they are connected around the top of the hierarchical tree (i.e., around the magnitude of 0.4). This indicates that these genes distinguish themselves from the other genes in the resistance to the selected antimicrobials. In this project, the hierarchical clustering was used to identify these antimicrobial-resistance genes that are most involved in multiple-antimicrobial resistance in the NPDIP data for the selected six states. The hierarchical clustering was also used to identify the foodborne pathogens/meats that are mainly responsible for carrying the antimicrobial-resistance genes in each of the six states. In addition, the clustering was used to study the similarity of the selected six states from the perspective of antimicrobial-resistance genes, antimicrobial resistance of foodborne pathogens, and the meats that carry antimicrobial-resistance genes. 

## 3. Results

Data from the NPDIB mentioned in the Materials and Methods section were analyzed by PCA and hierarchical clustering in this section to identify: (1) the antimicrobials to which pathogens show most resistance in the six states; (2) the genes mostly involved in antimicrobial resistance; (3) the major pathogens carrying antimicrobial-resistance genes in the six states; and (4) the major meats carrying antimicrobial-resistance genes in the six states. 

### 3.1. Identification of Antimicrobials to Which Pathogens Show Most Resistance in States PA, MD, NY, NM, MN, and CA

**Figure 3:**
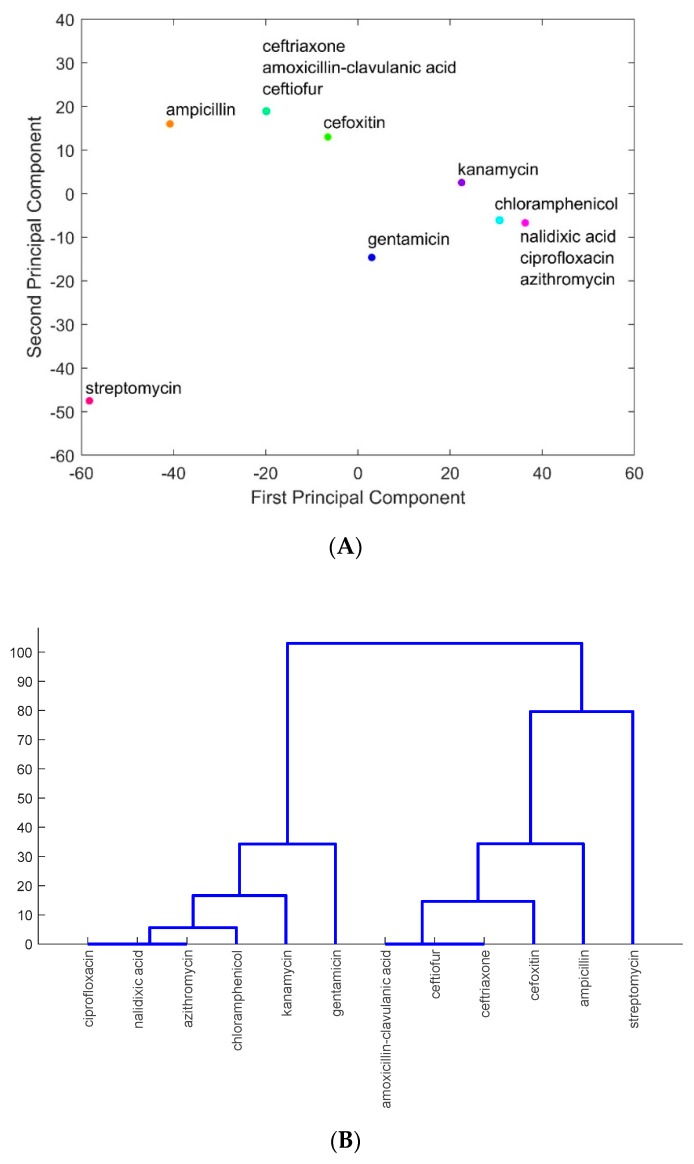
An illustrative example of hierarchical clustering: (**A**) projections of antimicrobials amoxicillin-clavulanic acid, ampicillin, azithromycin, cefoxitin, ceftiofur, ceftriaxone, chloramphenicol, ciprofloxacin, gentamicin, kanamycin, nalidixic acid, and streptomycin onto the PC1–PC2 space on the basis of the resistance of these antimicrobials in foodborne pathogens; (**B**) clustering the antimicrobials on the basis of their projections in (**A**).

The major antimicrobials to which foodborne pathogens isolated from meats in the six states were resistant were identified from PCA and from the hierarchical clustering as described in the section of Materials and Methods. As an example, Figure 3 shows the analysis results for the data from the state of Pennsylvania: Figure 3A projects the antimicrobials onto the PC1–PC2 space, and Figure 3B shows the results from hierarchical clustering. As seen in Figure 3A, streptomycin, gentamicin, ampicillin, kanamycin, and cefoxitin stand out from other antimicrobials as the ones getting most resistance by foodborne pathogens, including the species of *Campylobacter*, *Escherichia*, *Klebsiella*, *Legionella*, *Listeria*, *Providencia*, *Salmonella*, *Serratia*, *Shigella*, and *Vibrio*. While other antimicrobials are lumped together in Figure 3A due to their similarity in pathogen resistance, they can be identified in Figure 3B through a detailed hierarchical illustration of the similarity of those antimicrobials having resistance by pathogens. The blue lines in Figure 3B indicate the clusters of antimicrobials lumped together in Figure 3A. In particular, chloramphenicol, nalidixic acid, azithromycin, and ciprofloxacin are lumped together in Figure 3A. They are in the same branch of the hierarchical tree in Figure 3B. Similarly, amoxicillin-clavulanic acid, ceftiofur, and ceftriaxone are lumped together in Figure 3A.

The antimicrobials that stand out as the most resistant from the analysis of PCA and hierarchical clustering for all the six states are listed in Table 1. It can be seen that: (1) PA, NY, and MD have similarly resisted antimicrobials, including ampicillin, streptomycin, gentamicin, and kanamycin; (2) PA is the only state that does not show strong resistance to sulfisoxazole and tetracycline; (3) CA is the only state showing strong resistance to ciprofloxacin; (4) MN has the least number of the most resisted antimicrobials, and is the only one state that does not show strong resistance to gentamicin; and (5) ampicillin and streptomycin are the two common antimicrobials to which all six states show resistance. The eight antimicrobials shown in Table 1 contain five protein-synthesis inhibitors (i.e., streptomycin, tetracycline, chloramphenicol, gentamicin, and kanamycin), two cell-wall-synthesis inhibitors (i.e., ampicillin and cefoxitin), one competitive enzyme inhibitor (i.e., sulfisoxazole), one broad-spectrum antimicrobial (i.e., ciprofloxacin) that blocks cell division by inhibiting DNA gyrase, and a type II topoisomerase, topoisomerase IV.

### 3.2. Identification of Antimicrobial-Resistance Genes Most Common in States PA, MD, NY, NM, MN, and CA

**Figure 4:**
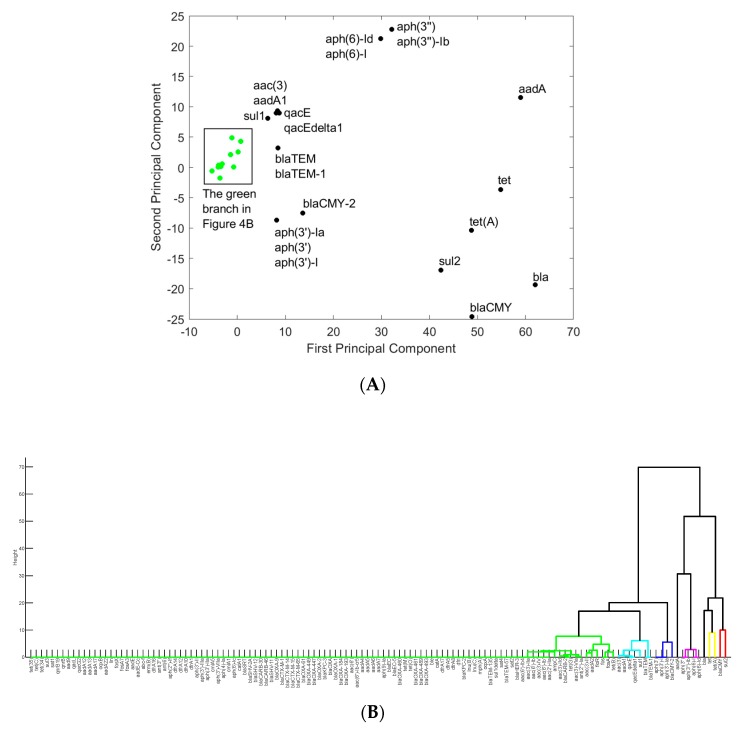
The representation of antimicrobial-resistance genes in the space of principal components one and two (**A**) and in the hierarchical clusters (**B**) for Pennsylvania. The genes shown in Green in (**A**) are listed in the Green branch in (**B**).

In order to identify the genes that are most involved in antimicrobial resistance in each of the six states, the data matrix was organized in such a way that each row represents one gene, while each column corresponds to an antimicrobial. As in Section 3.1, the data for PA were used as an example to illustrate the results obtained for a single state. The projection of the genes onto the PC1–PC2 space was shown in Figure 4A, while the hierarchical clustering of the genes was shown in Figure 4B. Genes *aadA*, *aph(3’’)*, *aph(3’’)-Ib*, *aph(6)-I*, *aph(6)-Id*, *bla*, *blaCMY*, *sul2*, *tet*, and *tet*(A) stand out from other genes as they are involved in resistance to a larger spectrum of antimicrobials than other genes. These genes are located separately in a single branch in Figure 4B. 

Antimicrobial resistance is typically caused by a group of synergistic genes. The group of genes that stand out from others in the hierarchical clustering diagram (Figure 4B) provides a potential set of synergistic genes that cause resistance to various antimicrobials in PA. A similar approach was applied to the data for the other five states, and the corresponding groups of genes were shown in Table S1 in the Supplementary Data section. The metabolic functions of these genes are listed in Table S2 in the Supplementary Data section: seven of them are involved in aminoglycoside resistance (including *aadA*, *aph(3′)*, *aph(3’)-Ia*, *aph(3”)*, *aph(3”)-Ib*, *aph(6)-I*, and *aph(6)-Id*); five of them are beta-lactamases (including *bla*, *blaCMY*, *blaCMY-2*, *blaTEM*, and *blaTEM-1*); three of them are tetracycline-resistance proteins (including *tet*, *tet(A)*, and *tet(B)*); and others are related to fosfomycin resistance (*fos* and *fos(A)*), efflux pump membrane transport (*oqxB*), and sulfate transmembrane transport (*sul2*). It is interesting to see that certain antimicrobial-resistance genes are shared among all six states (shown in Table 2). Among the six states, PA and NY have the same common antimicrobial-resistance genes, along with being very similar to MD, another nearby state. CA and MN notably lack *sul2*, NM is the only one to lack *tet*, and CA is the only one to lack *blaCMY*. 

### 3.3. Identification of Foodborne Pathogens Mostly Carrying Antimicrobial-Resistance Genes in PA, MD, NY, NM, MN, and CA

**Figure 5:**
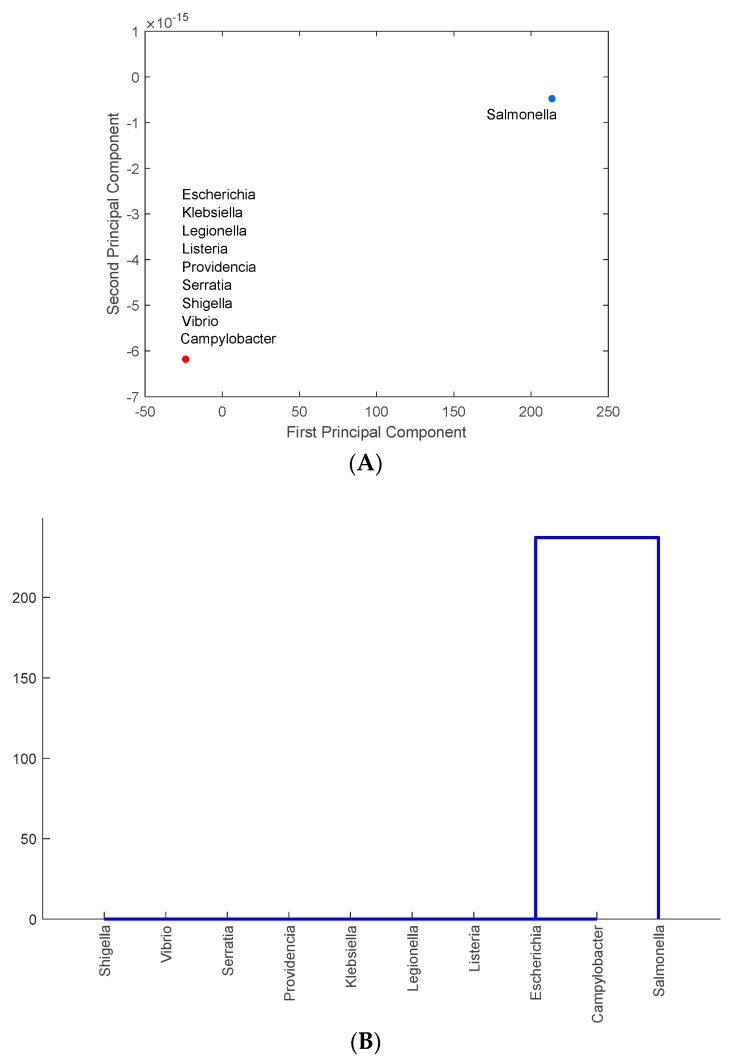
The representation of the major foodborne pathogens that carry antimicrobial-resistance genes in the PC1–PC2 space (**A**) and in the hierarchical clusters (**B**) for Pennsylvania.

It is important to identify the foodborne pathogens that carry the most common antimicrobial-resistance genes in each state. PCA and clustering approaches similar to those shown in the previous two sections were used to analyze foodborne pathogen and antimicrobial-resistance gene data. The data for PA were used here to illustrate results obtained for each state. Figure 5A shows the projection of the foodborne pathogens identified in PA onto the PC1–PC2 space, while Figure 5B illustrates the similar hierarchy of those pathogens carrying antimicrobial-resistance genes. Figure 5A shows that Salmonella stands out as the pathogen carrying more antimicrobial-resistance genes than the other 9 pathogens (which are lumped together in Figure 5A). Salmonella was found as the major carrier of antimicrobial-resistance genes in all the other five states. There are several different strains of Salmonella that may have different resistant genes. This can be further examined in future studies. MN has the smallest number of cases of Salmonella. This may be correlated with the smallest number of pathogen-resistant antimicrobials found in MN (as shown in Table 1). Slightly different from the other five states, California also has *Klebsiella* as another major antimicrobial-resistant foodborne pathogen (results not shown but similar to those in Figure 5B). 

### 3.4. Identification of Meats Mostly Carrying Antimicrobial-Resistance Genes in States PA, MD, NY, NM, MN, and CA

**Figure 6:**
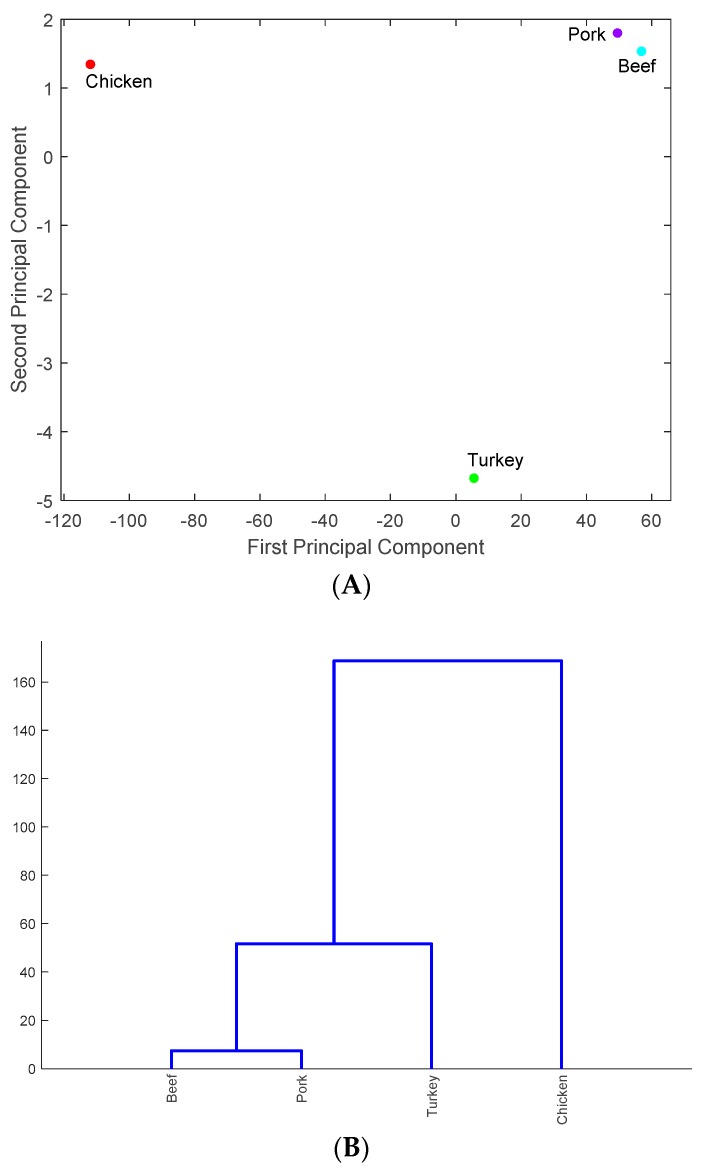
The representation of the meats that carry antimicrobial-resistance genes in the PC1–PC2 space (**A**) and in the hierarchical clusters (**B**) for Pennsylvania.

As in previous sections, the data for PA were used to illustrate the result of identifying the meats that carry antimicrobial-resistance genes in the six states. The four types of meats were projected onto the PC1–PC2 space in Figure 6A and the similarity of these meats carrying antimicrobial-resistance genes is shown in Figure 6B. As shown in Figure 6A, chicken and turkey are the two major meats that carry antimicrobial-resistance genes in PA. Similar results were obtained for the other five states. This is interesting, as CA, MN, and NM are not geographically close to each other or to the three eastern states PA, NY and MD. This may imply that meats with antimicrobial-resistance genes are distributed nationwide. In particular, poultry farms are mainly located in the eastern half of the US, but meats which carry antimicrobial-resistance genes may be being delivered to other regions in the US [17]. This mobility may explain why chicken and turkey were found to be the major carriers of antimicrobial-resistance genes in different regions of the US. 

## 4. Discussion

### 4.1. Overuse of Antimicrobials and Antimicrobial-Resistance Genes

Antimicrobial-resistance may be caused by the overuse of antimicrobials which drives the evolution of resistance [23]. This is also implied by the most commonly resisted antimicrobials that were identified in our results (i.e., ampicillin, streptomycin, gentamicin, kanamycin, cefoxitin, sulfisoxazole, tetracycline, and ciprofloxacin in Table 1) [24]. For example, the overuse of gentamicin triggers the resistance of *Enterococci*, which are isolated from pork in Michigan and chicken from Oregon [25]. While overuse of antimicrobials was found to be related to antimicrobial resistance, the use of antimicrobials in US food animal production has not been documented. There are no data indicating the dosages, duration, conditions, and locations for which antimicrobials are being used to raise different animals. The sales data of antimicrobials are typically used to indicate the trends in antimicrobial consumption in food animal production. Approximately 21.4 million pounds of medically important antimicrobials were sold for use in animal agriculture in 2015, a 26 percent total increase over 2009 sales [26]. The upward trend of sales of antimicrobials in animal agriculture correlates with increased antimicrobial resistance. 

Our results indicate that chicken and turkey are the two major meats that carry antimicrobial-resistance genes. These results are consistent with the foodborne pathogen infection diseases reported in the US. For example, Consumer Reports tests of chicken in both 2006 and 2010 revealed widespread presence of antimicrobial-resistant pathogens in retail poultry products. More than two-thirds of chicken samples were contaminated with *Salmonella* and/or *Campylobacter*, and more than 60 percent of those bacteria were resistant to one or more antibiotics [27]. In 2011, ground turkey was linked to 136 illnesses and one death, all caused by a strain of *Salmonella* that was resistant to four different antimicrobials: ampicillin, streptomycin, tetracycline, and gentamicin. Some 36 million pounds of ground turkey were recalled [28]. The antimicrobial resistance found in chicken and turkey was associated with the overuse of antimicrobials in their production. For example, the FDA’s rough estimate, using 1999 data, is that use of fluoroquinolones in chickens resulted in over 11,000 people that year contracting a strain of the campylobacter illness that was resistant to fluoroquinolones, and which contributed to an unnecessarily severe disease [29]. 

### 4.2. The Impact of Geographic Location on the Distribution of Antimicrobial-Resistance Genes

Genes *aadA*, *aph(3’’)*, *aph(3’’)-Ib*, *aph(6)-I*, *aph(6)-Id*, *bla*, *blaCMY*, *tet*, *tet*(A), *sul2* were identified from the historical data of the six states as the most common genes involved in the reported antimicrobial-resisted cases (Table 2). All of these genes are commonly found with antimicrobial resistance in Pennsylvania and New York, while certain genes are not commonly found with antimicrobial resistance in Maryland (*bla*), New Mexico (*bla* and *tet*), Minnesota (*sul2*), and California (*blaCMY* and *sul2*). It is interesting to see that Pennsylvania, New York, and Maryland, which are geographically close, share similar antimicrobial-resistance genes. This implies that the geographic location may affect the distribution of antimicrobial-resistance genes. On the other hand, Minnesota shares quite similar resistant genes with Pennsylvania and New York, even as it is geographically far from these two states. Similarly, California and New Mexico share eight of the common antimicrobial-resistance genes with Pennsylvania and New York. These findings indicate that geographic distance cannot limit the distribution of antimicrobial-resistance genes. One potential reason is that chicken and turkey, the two major meats identified by our results to carry antimicrobial-resistance genes, can be shipped nationwide. As mentioned in Section 3.4, poultry farms are mainly located in the eastern half of the US, but they deliver their products to other regions in the US. In this work, we focused on data reporting the antimicrobial-resistance genes carried by the four types of meats (i.e., chicken, turkey, pork, and beef). It is, thus, not surprising to find that these common antimicrobial-resistance genes can be spread from Pennsylvania, New York, and Maryland to other states. Another potential reason to explain why the six states share similar common antimicrobial-resistance genes is that similar antimicrobials are used in these states. Our results show that ampicillin, streptomycin, gentamicin, kanamycin, cefoxitin, sulfisoxazole, tetracycline, and ciprofloxacin are the major antimicrobials to which pathogens in the six states are resistant. The overuse of these antimicrobials in the animal production process imposes evaluation stress on pathogens, especially the major pathogen identified in our results, Salmonella. Antimicrobial-resistance genes from the pathogens surviving from the antimicrobial treatment are then spread to other microorganism species via horizontal gene transfer, and are then carried with the animal (meats) to other locations. 

### 4.3. Limitations and Future Work

Pathogens surviving from the treatment of antimicrobials generally carry multiple antimicrobial-resistance genes. It is, thus, important to find the set of antimicrobial-resistance genes that facilitate the survival of common foodborne pathogens despite treatment of various antimicrobials. We have identified this set of antimicrobial-resistance genes, including *aadA*, *aph(3’’)*, *aph(3’’)-Ib*, *aph(6)-I*, *aph(6)-Id*, *bla*, *blaCMY*, *tet*, *tet*(A), and *sul2* from data from six target states. The six states were selected to represent the northern, western, southern, and eastern regions of the US. Another reason for selecting these six states is that the NPDIB database contains significant data for them. While the findings of this work are limited to the six states, we will confirm our findings by analyzing data from additional states. One hurdle is the lack of NPDIB data for all US states; only a handful of states have more than 1000 data samples. 

## 5. Conclusions

The NPDIB database collects antimicrobial-resistant data sampled from foodborne pathogens in animal meats across the US. In this work, we presented the first multivariate statistical analysis to project antimicrobial-resistance gene data sampled from four types of meats (i.e., chicken, turkey, pork, and beef) for six states (i.e., PA, NY, MD, NM, MN, and CA) onto a two-dimensional space, thereby identifying the major antimicrobials, foodborne pathogens, genes, and meats involved in antimicrobial resistance. The results indicate that: 1) *aadA*, *aph(3’’)*, *aph(3’’)-Ib*, *aph(6)-I*, *aph(6)-Id*, *bla*, *blaCMY*, *tet*, *tet*(A), and *sul2* are the ten genes most found in antimicrobial-resistant foodborne pathogens; 2) these genes were mainly carried by Salmonella species in chicken and turkey; and 3) ampicillin, streptomycin, gentamicin, kanamycin, cefoxitin, sulfisoxazole, tetracycline, and ciprofloxacin are the major antimicrobials to which foodborne pathogens are resistant. While geographically adjacent states PA, NY, and MD share more similar antimicrobial-resistance genes than the others (i.e., MN, NM, and CA), all six states share common antimicrobial-resistance genes. This is likely explained by the finding that chicken and turkey, the two major meats that carry antimicrobial-resistance genes, are delivered nationwide. Overuse of antimicrobials in chicken and turkey were reported. This may explain why most antimicrobial-resistance genes were found in these two meats. Antimicrobial resistance is typically caused by the synergistic cooperation of multiple genes. The ten genes identified in this work (i.e., *aadA*, *aph(3’’)*, *aph(3’’)-Ib*, *aph(6)-I*, *aph(6)-Id*, *bla*, *blaCMY*, *tet*, *tet*(A), and *sul2*) provide valuable insight on this issue that may be used for future investigation. While the findings presented in this work are mainly based upon the antimicrobial-resistant data of foodborne pathogens for six states, they will be further validated and updated when the data for other states are more complete in the NPDIB database.

## Figures and Tables

**Table 1:** Antimicrobials to which pathogens are most resistant (indicated by PCA and hierarchical clustering for states PA, NY, MD, NM, MN, and CA).

	States	PA	NY	MD	NM	MN	CA
Antimicrobials							
Ampicillin							
Streptomycin							
Gentamicin							
Kanamycin							
Cefoxitin							
Sulfisoxazole							
Tetracycline							
Ciprofloxacin							

Note: the shaded areas indicate the resistance of antimicrobials in the corresponding states.

**Table 2:** The most common antimicrobial-resistance genes shared by the six states.

States	*aadA*	*aph(3’’)*	*aph(3’’)-Ib*	*aph(6)-I*	*aph(6)-Id*	*bla*	*blaCMY*	*tet*	*tet(A)*	*sul2*
PA										
NY										
MD										
NM										
MN										
CA										

Note: the shaded areas indicate involvement of the genes in the corresponding states.

## Supplementary Materials

The following are available online at https://www.mdpi.com/1660-4601/16/10/1811/s1, Table S1. Genes with most resistance to antimicrobials and hierarchical clustering, Table S2. Metabolic functions of the most common antimicrobial-resistance genes.
