# Supplementary material for: Data-Driven Analysis of Antimicrobial Resistance in Foodborne Pathogens from Six States within the US
Source: Int J Environ Res Public Health. 2019 May 22;16(10):1811. doi: 10.3390/ijerph16101811 (PMC6572035; doi:10.3390/ijerph16101811)
Supplement: Supplementary file 1 [file ijerph-16-01811-s001.pdf]

Table S1. Genes with most resistance to antimicrobials and hierarchical clustering.

States	Antimicrobial-resistance genes
PA	*aadA, aph(3′′), aph(3′′)-Ib, aph(6)-I, aph(6)-Id, bla, blaCMY, sul2, tet*, and *tet(A)*
NY	*aadA, aph(3′′), aph(3′′)-Ib, aph(3′), aph(3′)-Ia, aph(6)-I, aph(6)-Id, blaCMY, blaCMY-2, blaTEM, blaTEM-1, bla, sul2, tet*, and *tet(A)*
MD	*aadA, aph(3′′), aph(3′′)-Ib, aph(6)-I, aph(6)-Id, blaCMY, sul2, tet*, and *tet(A)*
NM	*aadA, aph(3′′)-Ib, aph(6)-I, aph(6)-Id, aac(3), aadA1, aph(3′), aph(3′)-Ia, blaCMY*, and *blaCMY-2, blaTEM, blaTEM-1, fos, fosA, qac, qacEdelta1, sul1, sul2, tet(A)*, and *tet(B)*
MN	*aadA, aadA1, aph(3′), aph(3′)-I, aph(3′′), aph(3′′)-Ib, aph(6)-I, aph(6)-Id, bla*, and *blaCMY, blaCMY-2, blaTEM, blaTEM-1, fosA, sul2, tet, tet(A)*, and *tet(B)*
CA	*aadA, aph(3′′), aph(3′′)-Ib, aph(6)-I, aph(6)-Id, bla, fos, fosA*, and *oqxB*

Table S2. Metabolic functions of the most common antimicrobial-resistance genes.

Genes	Metabolic Functions
aadA	• Aminoglycoside resistance<br>• Integration of the plastome-specific *aadA* cassette into the nuclear genome for a fraction of the resistant cell lines
aph(3′)	• Aminoglycoside resistance<br>• Catalysis of the addition of phosphate from ATP to the 3′-hydroxyl group of a 4,6-disubstituted aminoglycoside<br>• Origination from enzymes from the metabolic pathway for aminoglycosides and development in order to counteract the toxic effects of these antibiotics in the host bacterial cell<br>• Transferase
aph(3′)-Ia	• Aminoglycoside resistance<br>• A transposon-encoded aminoglycoside phosphotransferase<br>• Conference of resistance to kanamycin and neomycin
aph(3″)	• Aminoglycoside resistance<br>• Phosphorylation of streptomycin on the hydroxyl group at position 3′′
aph(3″)-Ib	• Aminoglycoside resistance<br>• Catalysis of ATP-dependent phosphorylation of a hydroxyl group<br>• Aminoglycoside phosphotransferase encoding by plasmids, transposons, integrative conjugative elements, and chromosomes in *Enterobacteriaceae* and *Pseudomonas* spp
aph(6)-I	• Aminoglycoside resistance<br>• Catalysis of ATP-dependent phosphorylation of a hydroxyl group
aph(6)-Id	• Aminoglycoside resistance<br>• Streptomycin phosphotransferase<br>• Phosphotransferase activity, alcohol group as an acceptor
bla	• Hydrolysis of the beta-lactam bond in susceptible beta-lactam antibiotics, thus conferring resistance to penicillin and cephalosporin

	• Beta-lactamase
blaCMY	• Hydrolysis of the beta-lactam bond in susceptible beta-lactam antibiotics<br>• ampC-related *bla* gene
blaCMY-2	• Hydrolysis of beta-lactam bond<br>• Beta-lactamase
blaTEM, *blaTEM-1*	• Hydrolysis of beta-lactam bond<br>• Responsibility of amino acid substitutions for the extended-spectrum beta lactamase (ESBL) phenotype cluster around the active site of the enzyme and change its configuration, allowing access to oxyimino-beta-lactam substrates.
tet, *tet(A)*, *tet(B)*	• Tetracycline-resistant protein<br>• Active tetracycline efflux<br>• Decrease of the accumulation of the antibiotic in whole cells<br>• Metal-tetracycline/H+ antiporter
fos, *fos(A)*	• Fosfomycin-resistant genes<br>• Inactivation of fosfomycin by addition of a glutathione residue
oqxB	• Efflux pump membrane transporter<br>• Component of RND-type multidrug efflux pump that confers resistance to olaquindox
sul2	• Dihydropteroate synthase activity<br>• High-affinity sulfate permease<br>• Sulfate transmembrane transporter activity
